# Physical Activity Across Adulthood and Physical Performance in Midlife: Findings from a British Birth Cohort

**DOI:** 10.1016/j.amepre.2011.06.035

**Published:** 2011-10

**Authors:** Rachel Cooper, Gita D. Mishra, Diana Kuh

**Affiliations:** a MRC Unit for Lifelong Health and Ageing, Division of Population Health, University College London, London, United Kingdom; b School of Population Health, University of Queensland, Herston, Australia

## Abstract

**Background:**

Evidence, mainly from cross-sectional studies, suggests that physical activity is a potentially important modifiable factor associated with physical performance and strength in older age. It is unclear whether the benefits of physical activity accumulate across life or whether there are sensitive periods when physical activity is more influential.

**Purpose:**

To examine the associations of leisure-time physical activity across adulthood with physical performance and strength in midlife, and to test whether there are cumulative benefits of physical activity.

**Methods:**

Using data on approximately 2400 men and women from the UK Medical Research Council National Survey of Health and Development, followed up since birth in March 1946, the associations of physical activity levels during leisure time self-reported prospectively at ages 36, 43, and 53 years with grip strength, standing balance, and chair rise times, assessed by nurses at age 53 years (in 1999), were examined in 2010.

**Results:**

There were independent positive effects of physical activity at all three ages on chair rise performance, and at ages 43 and 53 years on standing balance performance, even after adjusting for covariates. These results were supported by evidence of cumulative effects found when using structured life course models. Physical activity and grip strength were not associated in women and, in men, only physical activity at age 53 years was associated with grip strength.

**Conclusions:**

There are cumulative benefits of physical activity across adulthood on physical performance in midlife. Increased activity should be promoted early in adulthood to ensure the maintenance of physical performance in later life.

## Introduction

Maintaining physical performance and muscle strength with age is important given that lower levels in older populations are associated with increased risk of subsequent health problems, loss of independence, and shorter survival times.^1–3^ As the global population ages, there is a growing need to identify modifiable factors across life that influence physical performance and strength in later life. Such factors may influence the peak achieved in earlier life or the timing and rate of subsequent decline.^4,5^ It is therefore necessary to elucidate whether the effects of these factors accumulate across life or are more influential during sensitive periods when intervention to maintain or improve performance and strength is likely to be most beneficial.

Observational and intervention studies suggest that physical activity is a potentially important modifiable factor associated with physical performance and strength in older age.^6–18^ However, many of the existing observational studies are cross-sectional, so findings could be explained by reverse causality. In addition, few studies have examined the effect of physical activity earlier in life on physical performance and strength in mid to late adulthood and those that have are limited by the retrospective ascertainment of prior physical activity levels.^12^ It is therefore unclear whether the beneficial effects of physical activity accumulate over life or whether there are sensitive periods when physical activity is more beneficial.

Using data from a British birth cohort, the aim of the present study was to examine whether leisure-time physical activity levels at three ages across adulthood were associated with physical performance and strength in midlife. Another aim was to examine whether there was evidence of cumulative benefits of physical activity across adulthood or of sensitive periods when the impact of physical activity was greater than during other periods.

## Methods

The Medical Research Council National Survey of Health and Development (NSHD) is a socially stratified sample of all births that occurred during 1 week in March 1946 across England, Scotland, and Wales. This cohort of 5362 men and women has been followed up prospectively over 20 times across life from birth onwards. In 1999, when study participants were aged 53 years, 3035 were contacted successfully, of whom 2984 received a home visit from a nurse and 2956 successfully completed at least one of the physical performance or strength tests. Of those 2327 participants not successfully contacted in 1999, a total of 469 had died (8.7% of the original cohort); 948 had refused to participate (17.7%); 580 were abroad (10.8%); and 330 could not be traced (6.2%).^19^ The survey collects data on many aspects of health and lifestyle, including physical activity. The data collection in 1999 received ethical approval from the UK Multicentre Research Ethics Committee (MREC), and informed consent was given by participants to each set of questions and measures undertaken.

### Ascertainment of Physical Performance and Strength

Physical performance and strength were assessed during the home visits at age 53 years using three objective measures: grip strength, chair rises, and standing balance. Trained nurses conducted these tests using standardized protocols as described elsewhere.^20^

In summary, grip strength was measured isometrically using an electronic handgrip dynamometer.^21^ Two values were recorded for each hand and the highest were used in analyses. Chair rise time was measured as the time taken to rise from a sitting to a standing position with straight back and legs and then sit down again as fast as possible ten times. For high scores to indicate good performance, the reciprocal of the time taken (multiplied by 100) was used. Standing balance time was measured as the longest time, up to a maximum of 30 seconds, for which participants could maintain a one-legged stance in a standard position with their eyes closed. The distribution of these times was positively skewed and so they were normalized using a natural logarithm transformation (ln(seconds)).

### Ascertainment of Physical Activity Levels

Physical activity levels were ascertained at ages 36, 43, and 53 years during interviews with nurses at the study participants' homes. Different measures for physical activity were included in the surveys administered at the respective ages. At age 36 years, questions were asked about the frequency and duration of participation in 27 different leisure-time activities in the preceding month, based on the Minnesota leisure-time physical activity questionnaire.^22,23^ At age 43 years, participation in any sports, vigorous leisure activities, or exercises and how many months in the year and how often in these months each of the activities was done were reported. At age 53 years, participation in any sports, vigorous leisure activities or exercises in study participant's spare time, not including getting to and from work, in the past 4 weeks and the number of occasions on which these activities were undertaken was reported. At each age, participants were categorized as inactive (reported no participation); moderately active (participated in relevant activities one to four times: in the previous month at age 36 years, per month at age 43 years, and in the previous 4 weeks at age 53 years); or most active (participated in relevant activities five or more times: in the previous month at age 36 years, per month at age 43 years, and in the previous 4 weeks at age 53 years).

### Covariates

Factors that could confound the main associations were identified a priori. Height (cm) and weight (kg) were measured by nurses during the home visit at age 53 years. Own occupational class at age 53 years (or if not available, the most recent measure in adulthood [*n*=74]) was categorized using the Registrar General's Social Classification into three groups: I or II (high); IIINM or IIIM (medium); IV or V (low). Educational level attained at age 26 years was categorized into five groups: (1) degree or higher; (2) A levels, usually attained at age 18 years, or their equivalents; (3) O levels, usually attained at age 16 years, or their equivalents; (4) certificate of secondary education, clerical course, or equivalent; and (5) none. Health status at age 53 years was coded as a binary variable and identified those who reported being diagnosed with one or more of the following health conditions in the previous 10 years: diabetes, cancer, epilepsy, or cardiovascular disease (which was defined as having a heart attack or stroke ever, aortic stenosis or valvular disease in the past 10 years, doctor-diagnosed angina or Rose angina Grade I or II, or intermittent claudication). Smoking status at age 53 years was categorized as current, ex, or never smoker.

### Statistical Analyses

The associations between physical activity at each age and each of the physical performance and strength measures were tested using multiple linear regression models. In these and all subsequent models where there was evidence of an interaction between physical activity and gender, analyses were stratified by gender, but where not, analyses were gender-adjusted. Models were adjusted for current height and weight, then also for occupational class, educational level, smoking status, and health problems and finally also for physical activity at the other two ages with this final set of adjustments testing the independence of the effect of physical activity at each age from the effects of physical activity at other ages.

In a subsequent stage of analyses, at each of the three ages those classified as inactive were assigned a value of 0, those as moderately active a value of 1, and those as most active a value of 2. The scores from each age were then summed to create a lifetime physical activity score ranging from 0 (inactive at all three ages) to 6 (most active at all three ages). The association between this score (categorized into four groups: 0, 1 or 2, 3 or 4, and 5 or 6) and each of the outcomes was then tested with adjustments made for the same covariates as in previous models.

This model tested whether there were cumulative effects of physical activity across adulthood, assuming the effects of physical activity at each of the three ages were the same. To test whether an accumulation model such as this, an accumulation model that allows the size of the effect of physical activity at different ages to vary or a sensitive periods model best fit the data, the structured approach described by Mishra et al.^24^ then was applied. This involved comparing a series of nested models representing the two different accumulation models and a sensitive period model with a fully saturated model that assumes that all of the possible trajectories of physical activity across adulthood are associated with physical performance and strength (Appendix A, available online at www.ajpmonline.org). Large *p*-values indicate that the nested model fits the data as well as the saturated model and, therefore, that the hypothesis for the nested model is supported by the data. In these analyses, physical activity at each of the three ages were entered separately as linear ordinal terms with adjustments made for the same covariates as in previous models.

The analyses presented are not weighted and are based on the sample with complete data on physical activity at all three ages, all covariates and at least one of the outcome measures (*n*=2442). Analyses were also rerun (1) with inclusion of sample weights to allow for the stratified sampling design; (2) restricted to the sample who were inactive at age 53 years; and (3) on maximum available samples, but there were no differences in findings.

## Results

Men were stronger, had better physical performance levels at age 53 years, and were more likely to be active at ages 36 and 43 years than women (Table 1). Physical activity levels at each age were strongly associated with levels at the other two ages (*p*<0.01 from chi-square tests). Eighteen percent of the study participants were inactive, and 10% were most active at all three ages.

Physical activity levels at ages 36 and 43 years were not associated with grip strength (Table 2), and there was no evidence that these associations differed by gender (*p*=0.75 and 0.74 from tests of gender interaction, respectively). There was no association between physical activity at age 53 years and grip strength in women, but among men, those who were active at age 53 years had stronger grip strength than those who were inactive (*p*=0.01 from test of gender interaction), and this was maintained after adjustments. When using the structured approach, these findings were confirmed; the only nested model found to fit the data as well as the saturated model was that representing a sensitive period for physical activity at age 53 years among men (*p*=0.98).

There were graded associations between physical activity levels at all three ages and chair rise performance (Table 3) and no evidence of differences in association by gender (*p*-values from tests of gender interaction ≥ 0.30). These associations were maintained after adjustments. When the effects of physical activity at each of the three ages were adjusted mutually for each other, the associations of physical activity at each age were shown to be largely independent of each other.

Physical activity levels at all three ages were positively associated with standing balance (Table 4), with no evidence of differences in associations by gender (*p*-values from tests of gender interaction ≥ 0.09; Table 4). These associations were maintained after adjustments. When the effects of physical activity at each of the three ages were adjusted mutually for each other, the associations with standing balance performance were maintained for physical activity at ages 43 years and 53 years whereas the association of physical activity at age 36 years with balance attenuated.

Graded associations of the lifetime physical activity score with chair rise and standing balance performance were found (Table 5) suggesting that the benefits of physical activity for chair rise and standing balance performance are cumulative across adulthood. This was supported by the findings when using the structured approach that showed that the only nested models to fit the data as well as the fully saturated model were an accumulation model, assuming similar effect sizes at each age (*p*=0.48), for chair rises and an accumulation model, allowing for differences in effect size at each age (*p*=0.23), for standing balance.

## Discussion

In a nationally representative British population, evidence was found of cumulative benefits of physical activity across adulthood for physical performance in midlife. These associations were robust to adjustment for a range of potential confounding factors. There was also evidence to suggest that higher current physical activity levels were associated with stronger grip strength in men.

The results with respect to physical performance support other study findings^9,12^ and extend the existing literature by providing evidence of the cumulative benefits of physical activity across adulthood. The most consistent evidence of a cumulative effect of physical activity was found in relation to chair rising. This could be due to the fact that the types of leisure-time physical activity that NSHD study participants undertake are beneficial for lower body strength and power, essential components of good performance in the chair rising test. Likewise, high levels of lifetime physical activity have a positive impact on cardiorespiratory fitness,^25,26^ which is required to successfully complete the chair rising test. It is also possible that the association operates through body weight; while the association was maintained after adjustment for current weight there could be residual confounding by lifetime weight change. In the NSHD, both standing balance and chair rising ability are more strongly associated than grip strength with neuromuscular speed and control and cognitive performance,^27^ which may be influenced by lifetime physical activity.

The current study is not the only one to find inconsistent evidence of effects of physical activity on strength.^11,28^ However, some observational studies^10,15,16^ have found associations between physical activity and grip strength among women. The findings in this paper may be in contrast to other studies because of the younger age of the sample. Differences in findings could be also due to differences in the types of activity undertaken.

Intervention studies^13,26,29^ demonstrating a beneficial effect of physical activity on strength have implemented specific training regimes designed to improve strength and performance which may not be equivalent to the type of physical activity generally undertaken. These results suggest that among the NSHD participants, recreational physical activity of the correct type, intensity, or frequency to beneficially affect upper body strength, as demonstrated in trials,^26^ is not being taken sufficiently often especially among women. Men are more likely than women to undertake leisure-time activities such as racquet sports, martial arts, and weight training^30^ that improve and maintain their upper body strength; this may explain the gender difference in association that was found.

A strength of the present study is the prospective collection of information on physical activity at multiple times across adulthood. Although these measures were self-reported, they have each been found to be correlated in expected directions with other health behaviors and BMI. Further, the Minnesota leisure-time physical activity questionnaire^22^ on which the assessment at age 36 years was based was shown to have a high 1-month reliability and was correlated with a treadmill estimation of oxygen uptake and body composition.^31^ Nonetheless, improvements in objective measures of physical activity will be needed to validate these relationships.

Another strength is the availability of objective measures of physical performance and strength. These are expected to be less subject to bias than self-reported measures used in some other studies.^8^ Assessing these outcomes in midlife makes it less likely that findings will be explained by comorbidities. In older populations, these are likely to have an impact on physical activity as well as performance and strength and to confound associations.

To enable physical activity across adulthood to be examined, participation in sports, recreational, and leisure-time activities was the main explanatory factor selected as information on this had been ascertained at all three ages. It is acknowledged that this information was not ascertained using the same instrument at each age; however, these measures are associated. Further, these measures do not take into consideration work time, active travel, or domestic physical activity. However, the amount of activity that people undertake as part of their daily lives is decreasing, and participation in leisure-time activity is increasingly necessary to ensure that the recommended levels of physical activity are met.^32,33^ Leisure-time physical activity is therefore likely to be a reasonably representative indicator of overall physical activity levels and an important target for intervention.

The NSHD cohort was established using a sampling frame that ensured that it was nationally representative of the population born in England, Scotland, and Wales in 1946. Since then, losses to follow-up due to death, emigration, loss of contact, and permanent refusal have occurred. Despite this, at age 53 years, the sample remained representative of the national population born at a similar time in most respects,^19,34^ and so findings should be generalizable to the generation of postwar baby boomers currently reaching old age. In addition, 514 of the 2956 people with at least one valid outcome measure were excluded from analyses because of missing data.

When the characteristics of those excluded were compared with those included, there were no differences in the distributions of most key characteristics (Appendix B, available online at www.ajpmonline.org). However, those excluded were more likely to be inactive at age 53 years, have no educational qualifications, and be current smokers than those included. It is not expected that these differences would introduce substantial bias. There was also a small proportion of study participants who were unable to perform one or more of the outcome assessments (grip strength *n*=69, chair rising *n*=154, standing balance *n*=113). These people were more likely to have health problems, poorer performance on the other tests, and a lower educational level (results not shown). Those people unable to perform the chair rise and standing balance tests were also more likely to be inactive, so by excluding these people the associations found may be weaker than they would have been if these individuals had been included.

The findings in relation to chair rising and standing balance performance suggest that promotion of leisure-time physical activity across adulthood would have beneficial effects on physical performance later in life and hence the functional health and quality of life of the aging population, especially as the size of the differences in performance detected may be clinically relevant. Promotion of leisure-time activity is likely to become increasingly important in younger populations as people's daily routines become more sedentary.^32^ That not all people categorized as most active in the current study will meet the recommended level of at least 30 minutes of moderate-intensity physical activity five times a week^32^ (e.g., only approximately 35% of people in the most active group at age 53 years reported this frequency of activity) suggests that only low levels of physical activity need to be achieved for there to be beneficial effects on physical performance. Findings in relation to grip strength suggest that specific exercises and activities may need to be promoted to ensure that people undertake physical activity that is beneficial for upper body strength.

It has been proposed that the associations of physical performance and strength with mortality rates and cardiovascular disease^2,3,35^ may be explained by the fact that performance and strength are acting as markers of lifetime physical activity.^35,36^ The results with respect to physical performance provide support for this explanation but the findings with respect to grip strength suggest that this is unlikely to fully explain the grip strength–mortality associations.^2^

## Conclusion

Increased activity should be promoted early in adulthood to ensure the maintenance of physical performance in later life. When promoting physical activity, it may be necessary to encourage people to participate in specific types of activity in order for a beneficial effect on upper body strength to be seen.

## Figures and Tables

**Table 1:** Characteristics of the sample (N=2442), *n* (%) unless otherwise specified

	Men (*n*=1189^a^)	Women (*n*=1253^a^)
**STRENGTH AND PHYSICAL PERFORMANCE AT AGE 53 YEARS** (M [SD])		
**Grip strength (kg)**	47.8 (12.0)	27.8 (8.0)
**Chair rise time ((1/s) ×100)**^b^	5.3 (1.7)	5.0 (1.6)
**Standing balance time (seconds)**^c^	5.5 (2.2)	4.5 (2.0)
**PHYSICAL ACTIVITY AT AGE**		
**36 years**		
Inactive	363 (30.5)	510 (40.7)
Moderately active	319 (26.8)	309 (24.7)
Most active	507 (42.6)	434 (34.6)
**43 years**		
Inactive	562 (47.3)	689 (55.0)
Moderately active	281 (23.6)	290 (23.1)
Most active	346 (29.1)	274 (21.9)
**53 years**		
Inactive	555 (46.7)	621 (50.0)
Moderately active	240 (20.2)	210 (16.8)
Most active	394 (33.1)	422 (33.7)
**OTHER CHARACTERISTICS AT AGE 53 YEARS**		
**Weight (kg; M [SD])**	83.5 (13.2)	71.8 (14.3)
**Height (cm; M [SD])**	174.6 (6.4)	161.7 (5.9)
**Occupational class**		
I or II (High)	614 (51.6)	457 (36.5)
III (Medium)	458 (38.5)	534 (42.6)
IV or V (Low)	117 (9.8)	262 (20.9)
**Educational level**		
Degree or higher	181 (15.2)	65 (5.2)
A levels or equivalent	341 (28.7)	299 (23.9)
O levels or equivalent	184 (15.5)	327 (26.1)
CSE, clerical course or equivalent	71 (6.0)	118 (9.4)
None	412 (34.7)	444 (35.4)
**Disabling/life-threatening health conditions**		
None	1058 (89.0)	1104 (88.1)
One or more	131 (11.0)	149 (11.9)
**Smoking status**		
Never	426 (35.8)	621 (50.0)
Ex	500 (42.1)	356 (28.4)
Current	263 (22.1)	276 (22.0)

*Note:* The sample includes those with data on covariates (Appendix C, available online at www.ajpmonline.org inlcudes comparison with sample with missing data on covariates), physical activity at all three ages, and at least one of the physical performance or strength measures. A levels are those usually attained at age 18 years; O levels are those usually attained at age 16 years. CSE, Certificate of Secondary Education

a Except for grip strength: men=1155, women=1205; chair rising: men=1119, women=1171; standing balance: men=1129, women=1182

b Reciprocal of time taken for 10 chair rises × 100 (e.g., a value of 5 = 20 seconds to complete 10 chair rises)

c Geometric M and SD

**Table 2:** Associations between physical activity levels across adulthood and grip strength at age 53 years (*n*=2360)

Physical activity at age	*n* (%)	Difference in mean grip strength (kg; 95% CI)		
		Model 1	Model 2	Model 3
**36 years**				
Inactive	843 (35.7)	0.00	0.00	0.00
Moderately active	606 (25.7)	0.07 (−0.97, 1.12)	0.02 (−1.03, 1.07)	−0.24 (−1.30, 0.82)
Most active	911 (38.6)	0.69 (−0.26, 1.63)	0.64 (−0.31, 1.60)	0.25 (−0.78, 1.27)
*p*-value^a^		0.30	0.33	0.66
**43 years**				
Inactive	1210 (51.3)	0.00	0.00	0.00
Moderately active	550 (23.3)	0.55 (−0.46, 1.56)	0.53 (−0.48, 1.55)	0.10 (−0.95, 1.14)
Most active	600 (25.4)	0.47 (−0.52, 1.46)	0.47 (−0.53, 1.48)	−0.27 (−1.36, 0.82)
*p*-value^a^		0.47	0.49	0.81
**53 years**				
**Men (*n*=1155)**				
Inactive	536 (46.4)	0.00	0.00	0.00
Moderately active	235 (20.4)	3.30 (1.49, 5.11)	3.35 (1.50, 5.20)	3.44 (1.53, 5.34)
Most active	384 (33.3)	2.53 (0.98, 4.08)	2.83 (1.24, 4.42)	2.95 (1.26, 4.64)
* p*-value^a^		<0.001	<0.001	<0.001
**Women (*n*=1205)**				
Inactive	596 (49.5)	0.00	0.00	0.00
Moderately active	199 (16.5)	−0.16 (−1.41, 1.09)	−0.30 (−1.58, 0.98)	−0.30 (−1.59, 0.99)
Most active	410 (34.0)	0.67 (−0.32, 1.67)	0.59 (−0.44, 1.62)	0.54 (−0.54, 1.62)
*p*-value^a^		0.31	0.34	0.41

*Note:* Model 1: adjusted for gender (if no evidence of gender interaction in unadjusted model), current height and weight; Model 2: Model 1 plus adult SEP (own occupation and education), smoking and health problems at age 53 years; Model 3: Model 2 plus physical activity levels at the other two ages (including gender × physical activity at age 53 years interaction). Inactive = no participation in relevant activities; moderately active = participated in relevant activities one to four times: in the previous month at age 36 years, per month at age 43 years, and in the previous 4 weeks at age 53 years; most active = participated in relevant activities five or more times: in the previous month at age 36 years, per month at age 43 years, and in the previous 4 weeks at age 53 years

a *p*-value from likelihood ratio test comparing a model with physical activity at specified age included to a model with physical activity not included

**Table 3:** Associations between physical activity levels across adulthood and chair rise performance at age 53 years (*n*=2290)

Physical activity at age	*n* (%)	Difference in mean reciprocal chair rise time (1/s ×100) (95% CI)		
		Model 1	Model 2	Model 3
**36 years**				
Inactive	797 (34.8)	0.00	0.00	0.00
Moderately active	592 (25.9)	0.22 (0.05, 0.39)	0.17 (−0.001, 0.34)	0.09 (−0.09, 0.26)
Most active	901 (39.3)	0.60 (0.44, 0.75)	0.52 (0.37, 0.68)	0.34 (0.18, 0.51)
*p*-value^a^		<0.001	<0.001	<0.001
**43 years**				
Inactive	1153 (50.4)	0.00	0.00	0.00
Moderately active	541 (23.6)	0.42 (0.25, 0.58)	0.37 (0.20, 0.53)	0.26 (0.09, 0.43)
Most active	596 (26.0)	0.64 (0.47, 0.80)	0.56 (0.39, 0.72)	0.35 (0.17, 0.52)
*p*-value^a^		<0.001	<0.001	<0.001
**53 years**				
Inactive	1067 (46.6)	0.00	0.00	0.00
Moderately active	437 (19.1)	0.37 (0.19, 0.55)	0.29 (0.10, 0.47)	0.18 (−0.01, 0.36)
Most active	786 (34.3)	0.55 (0.40, 0.70)	0.47 (0.32, 0.62)	0.29 (0.13, 0.45)
*p*-value^a^		<0.001	<0.001	0.002

*Note:* Model 1: adjusted for gender (if no evidence of gender interaction in unadjusted model), current height, and weight; Model 2: Model 1 plus adult SEP (own occupation and education), smoking and health problems at age 53 years; Model 3: Model 2 plus physical activity levels at the other two ages. Inactive = no participation in relevant activities; moderately active = participated in relevant activities one to four times: in the previous month at age 36 years, per month at age 43 years, and in the previous 4 weeks at age 53 years; most active = participated in relevant activities five or more times: in the previous month at age 36 years, per month at age 43 years, and in the previous 4 weeks at age 53 years.

a *p*-value from likelihood ratio test comparing a model with physical activity at specified age included to a model with physical activity not included

**Table 4:** Associations between physical activity levels across adulthood and standing balance performance at age 53 years (*n*=2311)

Physical activity at age	*n* (%)	Difference in mean ln(standing balance time [s]) (95% CI)		
		Model 1	Model 2	Model 3
**36 years**				
Inactive	822 (35.6)	0.00	0.00	0.00
Less active	596 (25.8)	0.10 (0.01, 0.18)	0.05 (−0.03, 0.13)	0.01 (−0.07, 0.09)
Most active	893 (38.6)	0.21 (0.14, 0.28)	0.14 (0.07, 0.21)	0.05 (−0.02, 0.13)
*p*-value^a^		<0.001	0.001	0.35
**43 years**				
Inactive	1166 (50.5)	0.00	0.00	0.00
Less active	553 (23.9)	0.24 (0.16, 0.32)	0.19 (0.12, 0.27)	0.16 (0.08, 0.24)
Most active	592 (25.6)	0.31 (0.23, 0.39)	0.24 (0.16, 0.32)	0.19 (0.11, 0.27)
*p*-value^a^		<0.001	<0.001	<0.001
**53 years**				
Inactive	1092 (47.3)	0.00	0.00	0.00
Less active	435 (18.8)	0.25 (0.16, 0.33)	0.17 (0.09, 0.26)	0.13 (0.04, 0.21)
Most active	784 (33.9)	0.23 (0.16, 0.30)	0.15 (0.08, 0.23)	0.08 (0.01, 0.16)
*p*-value^a^		<0.001	<0.001	0.01

*Note:* Model 1: adjusted for gender (if no evidence of gender interaction in unadjusted model), current height and weight; Model 2: Model 1 plus adult SEP (own occupation and education), smoking and health problems at age 53 years; Model 3: Model 2 plus physical activity levels at the other two ages. Inactive = no participation in relevant activities; moderately active = participated in relevant activities one to four times: in the previous month at age 36 years, per month at age 43 years, and in the previous 4 weeks at age 53 years; most active = participated in relevant activities five or more times: in the previous month at age 36 years, per month at age 43 years, and in the previous 4 weeks at age 53 years.

a *p*-value from likelihood ratio test comparing a model with physical activity at specified age included to a model with physical activity not included

**Table 5:** Associations between lifetime physical activity score and physical performance at age 53 years

Lifetime physical activity score	Chair rise performance (*n*=2290)		Standing balance (*n*=2311)	
	*n* (%)	Difference in M (1/s ×100) (95% CI)	*n* (%)	Difference in M ln(s) (95% CI)
0	396 (17.3)	0.00	416 (18.0)	0.00
1–2	749 (32.7)	0.24 (0.04, 0.44)	744 (32.2)	0.07 (−0.02, 0.16)
3–4	676 (29.5)	0.52 (0.31, 0.72)	687 (29.7)	0.20 (0.10, 0.29)
5–6	469 (20.5)	0.93 (0.70, 1.15)	464 (20.1)	0.31 (0.21, 0.42)
*p*-value^a^		<0.001		<0.001

*Note:* Effect estimates presented are adjusted for: gender; current height and weight; adult SEP (own occupation and education); smoking; and health problems at age 53 years Lifetime physical activity score derived by assigning those classified as inactive a value of 0, those as moderately active a value of 1, and those as most active a value of 2 at each age and then summing the values for the three ages whereby an individual with a physical activity score of 0 has been categorized as inactive at all three ages, whereas an individual with a physical activity score of 6 has been categorized as most active at all three ages.

a *p*-value from likelihood ratio test comparing a model with the physical activity score included to a model with the score not included

## Supplementary data

Appendixes A to C
