# Supplementary material for: Physical Activity Across Adulthood and Physical Performance in Midlife: Findings from a British Birth Cohort
Source: Am J Prev Med. 2011 Oct;41(4):376–84. doi: 10.1016/j.amepre.2011.06.035 (PMC3185208; doi:10.1016/j.amepre.2011.06.035)
Supplement: Appendixes A to C [file mmc1.pdf]

Physical Activity Across Adulthood and Physical Performance in Midlife

Findings from a British Birth Cohort Study

Rachel Cooper, PhD, Gita D. Mishra, PhD, Diana Kuh, PhD

Appendix A

Models compared when using the structured life course modelling approach[1]

Fully saturated model:

$E(Y)= \alpha + \beta_1 Pa_1 + \beta_2 Pa_2 + \beta_3 Pa_3 + \theta_{12} Pa_1 Pa_2 + \theta_{23} Pa_2 Pa_3 + \theta_{13} Pa_1 Pa_3 + \theta_{123} Pa_1 Pa_2 Pa_3$

Compared with:

(1) Critical* period model

$E(Y)= \alpha + \beta_1 Pa_1$

constraints: $\beta_2= \beta_3=0$; $\theta_{12}=\theta_{23}=\theta_{13}=\theta_{123}=0$

(2) Accumulation model: summed score (assuming similar effect sizes at each age)

$E(Y)= \alpha + \beta \Sigma_j Pa_j$

constraints: $\beta_1= \beta_2=\beta_3$; $\theta_{12}=\theta_{23}=\theta_{13}=\theta_{123}=0$

(3) Accumulation model: mutually adjusted (allowing for differences in effect size at each age)

$E(Y)= \alpha + \beta_1 Pa_1 + \beta_2 Pa_2 + \beta_3 Pa_3$

constraints: $\beta_1 \neq \beta_2 \neq \beta_3$; $\theta_{12}=\theta_{23}=\theta_{13}=\theta_{123}=0$

Notes:

Pa=Physical activity; 1=at age 36 years; 2=at age 43 years; 3=at age 53 years

*Critical periods may be more evident for chronic disease risk associated with developmental mechanisms in biological subsystems, whereas sensitive periods are likely to be more common in behavioral development[2]

References for Appendix A

1. Mishra G, Nitsch D, Black S, De Stavola B, Kuh D, Hardy R. A structured approach to modelling the effects of binary exposure variables over the life course. Int J Epidemiol 2009;38:528–37.
2. Mishra GD, Ben-Shlomo Y, Kuh D. A life course approach to health behaviors: theory and methods. In: Steptoe A, ed. Handbook of Behavioral Medicine: Methods and Applications. New York: Springer, 2010.

Appendix B

Associations between lifetime physical activity score and physical performance at age 53 years

Lifetime physical activity score	Chair rise performance (*n*=2290)		Standing balance (*n*=2311)	
	n (%)	Difference in M (1/s*100) (95% CI)	*n* (%)	Difference in M ln(s) (95% CI)
0	396 (17.3)	0.00	416 (18.0)	0.00
1–2	749 (32.7)	0.24 (0.04, 0.44)	744 (32.2)	0.07 (-0.02, 0.16)
3–4	676 (29.5)	0.52 (0.31, 0.72)	687 (29.7)	0.20 (0.10, 0.29)
5–6	469 (20.5)	0.93 (0.70, 1.15)	464 (20.1)	0.31 (0.21, 0.42)
p-value[a]		<0.001		<0.001

Note: Effect estimates presented are adjusted for: gender, current height and weight, adult SEP (own occupation and education), smoking and health problems at age 53 years. Lifetime physical activity score derived by assigning those classified as inactive a value of 0, those as moderately active a value of 1, and those as most active a value of 2 at each age and then summing the values for the three ages whereby an individual with a physical activity score of 0 has been categorized as inactive at all three ages, whereas an individual with a physical activity score of 6 has been categorized as most active at all three ages.

[a] *p*-value from likelihood ratio test comparing a model with the physical activity score included to a model with the score not included

Appendix C

Characteristics of the sample included in analyses (*n*=2442) compared with the sample excluded due to missing data on covariates

	% or M (SD)		
	Excluded (*n*=514[a])	Included (*n*=2442)	*p*-value[b]
**Strength and physical performance at age 53 years**			
Grip strength (kg)	37.6 (14.5)	37.6 (14.3)	0.92
Chair rise time ((1/s)x100)[c]	5.2 (1.8)	5.2 (1.7)	0.99
Standing balance time (s)[d]	1.6 (0.8)	1.6 (0.8)	0.44
PHYSICAL ACTIVITY AT GIVEN AGE			
**36 years**			
Inactive	41.9	35.8	0.18
Moderately active	23.6	25.7	
Most active	34.5	38.5	
**43 years**			
Inactive	52.5	51.2	0.86
Moderately active	22.1	23.4	
Most active	25.4	25.4	
**53 years**			
Inactive	53.0	48.2	0.01
Moderately active	13.1	18.4	
Most active	33.9	33.4	
OTHER CHARACTERISTICS AT 53 YEARS			
**Gender (Male)**	51.2	48.7	0.31
**Weight (kg)**	77.6 (16.3)	77.5 (14.9)	0.89
**Height (cm)**	168.3 (9.5)	168.0 (8.9)	0.52
**Occupational class**			
I or II	41.3	43.9	0.11
III	39.4	40.6	
IV or V	19.3	15.5	
**Educational level**			
Degree or higher	8.5	10.1	0.002
A levels or equivalent	23.0	26.2	
O levels or equivalent	16.8	20.9	
CSE, clerical course or	5.7	7.7	
equivalent	46.0	35.1	
None			
**Disabling/life-threatening health conditions**			
None	87.2	88.5	0.38
One or more	12.8	11.5	
**Smoking status**			
Never	38.1	42.9	0.007
Ex	33.5	35.1	
Current	28.4	22.1	

[a] Total *n* varies due to missing data

[b] *p*-values from t-test or chi-squared test, as appropriate

[c] Reciprocal of time taken for 10 chair rises x 100 (for example, a value of 5=20s to complete 10 chair rises)

[d] Geometric M and SD
